# Oxidative Stress Assessment in Response to Ultraendurance Exercise: Thiols Redox Status and ROS Production according to Duration of a Competitive Race

**DOI:** 10.1155/2016/6439037

**Published:** 2016-07-18

**Authors:** Alessandra Vezzoli, Cinzia Dellanoce, Simona Mrakic-Sposta, Michela Montorsi, Sarah Moretti, Annamaria Tonini, Lorenza Pratali, Roberto Accinni

**Affiliations:** ^1^Institute of Bioimaging and Molecular Physiology, National Research Council (CNR), Via Fratelli Cervi 93, 20090 Segrate, Italy; ^2^Institute of Clinical Physiology, National Research Council (CNR), Niguarda Ca' Granda Hospital, Via G. Moruzzi 1, 56124 Pisa, Italy; ^3^Telematic University San Raffaele, Via F. Daverio 7, 20122 Milan, Italy

## Abstract

*Purpose*. Response to an ultraendurance competitive race on thiols redox status, reactive oxygen species (ROS) production, and oxidative stress (OxS) was investigated according to duration.* Methods*. Twenty-four elite runners were examined: six completed 50 km and eighteen 100 km. Blood and urine samples were collected before and immediately after the race. Erythrocytes and plasma aminothiols by high-performance liquid chromatography, total antioxidant capacity (TAC), and OxS biomarkers (protein carbonyl (PC), thiobarbituric acid-reactive substances (TBARS), 8-isoprostane (8-iso-PGF2*α*), and 8-OH-2-deoxyguanosine (8-OH-dG)) by immunoenzymatic assays and ROS production by Electron Paramagnetic Resonance were assessed.* Results*. Significant increases (*P* between <0.05 and <0.0001) were recorded in plasma total and oxidized aminothiols concentration and TAC (*P* < 0.0001) only after 100 km: plasmatic (ROS production (+12 versus +29%), PC (+54 versus +115%), and TBARS (+28 versus +55%)) and urinary (8-OH-dG.creatinine^−1^ (+71 versus +158%) and 8-iso-PGF2*α*.creatinine^−1^ (+43 versus +135%)) concentrations for 50 and 100 km (duration 4 h 3′ versus 8 h 42′), respectively.* Conclusion*. Very prolonged ultraendurance exercise causes an increase in ROS production and OxS depending on specific biomarker examined but always linearly and directly related to exercise duration. Redox status of erythrocytes was preserved. A relationship between running performance and both prerace ROS production and antioxidant-redox status was found in 100 km race.

## 1. Introduction

Despite the many known health benefits of exercise, there is a body of evidence suggesting that moderate/vigorous physical activity results in enhanced formation of reactive oxygen species (ROS). Over the past 20 years, perceptions on the roles of ROS have changed and are now considered physiologically vital in the process of signal transduction. Despite this, if they are not balanced or cleared, then they may cause structural damage to various macromolecules [1]. Electron Paramagnetic Resonance (EPR) is the only technique that provides direct evidence of the “instantaneous” presence of ROS leading to absolute concentrations. However, most adopted methods for assessing oxidative stress (OxS) utilize the measurement of ROS-induced modifications to proteins, DNA, and lipids. Adducts on these molecules, referred to as biomarkers, determined in blood correlate with similar measurements in tissues and therefore their assessment provides an indication of whole-body OxS [2].

The vast majority of the human studies have measured the redox status of plasma. This is probably done due to the easiness of plasma collection and because it is assumed that plasma better reflects tissue redox status. Indeed, plasma interacts with organs and tissues that are a possible source of ROS and the exchange from the tissues to the plasma is bidirectional. Moreover, ROS can be produced in plasma mainly through reactions with metals [3].

The activity/concentration of the antioxidants in blood greatly affects the extent of ROS accumulation. The extent of ROS production has the potential to draw on antioxidant defences [4]; therefore, measurement of these latter provides another informative method to assess OxS.

Aminothiols are the major nonenzymatic antioxidant compounds characterized by reduced sulfhydryl moieties that directly quench ROS and are present in all physiological samples (fluids and tissues). The most abundant in tissues is reduced glutathione (GSH) that serves multiple functions protecting from oxidative damage and keeping the intracellular environment in the reduced state [5]. Various other aminothiols, including homocysteine (Hcy), cysteine (Cys), and cysteinylglycine (CysGly), are metabolically strictly related and can be considered the principal interface between the changing redox environment and protein activity. In plasma, aminothiols interact via redox and disulfide exchange reactions generating a dynamic system referred to as redox thiol status which, regulating cellular homeostasis, is a critical determinant of cell function. Indeed, altered concentration of one aminothiol species causes complex changes in overall thiol dynamic equilibrium and in other biological mechanisms even to the disruption of the redox-regulated signaling mechanisms and any major imbalances can cause severe cellular damage or death [6]. Human plasma thiols redox status can be used as index of a systemic response of the organism and the detection and quantification of reduced and oxidized forms of aminothiols are important in the investigation of oxidative stress-related risk factors and diseases [7].

Aminothiols are the major nonenzymatic antioxidant compounds in erythrocytes too. These cells are particularly susceptible to oxidative insult due to their role as oxygen transporters and the high content of polyunsaturated fatty acids, transition metals, and redox active hemoglobin (Hb) molecules. Indeed, as the functional role of erythrocytes is the transport of oxygen bound to Hb, this induces continuous slow autoxidation of Hb producing superoxide [8]. However, erythrocytes are characterized by an extensive antioxidant capacity in order to protect the body from a potential major source of OxS, but exercise in extreme conditions, that is, when antioxidant compounds are depleted [9], may induce a so high OxS that their antioxidant protection diminishes. For all these reasons, erythrocytes have been used as a simple model (a) to study the cellular effects of various compounds [10], especially those that generate ROS, and (b) because they are appropriate for intracellular redox status analysis.

Strenuous exercise increases oxidant production by muscle, limiting performance during endurance exercise tasks. It was suggested that ultraendurance events, like long-term running, cause OxS to be associated with a period of reduced antioxidant protection [11]. A self-accelerating vicious cycle of ROS release from oxidative damaged tissue has been implicated in aging, neurodegenerative diseases, and apoptosis [12, 13]; consequently, it has been substantially debated whether the health benefits of physical activity extend to more extreme forms of endurance exercise [14, 15]. The level to which exposure to stressors is detrimental for the organism depends on the nature and strength of the stressor. Major factors that may affect oxidation response to exercise are type and intensity of the exercise. Many studies have investigated antioxidant status and OxS with subjects involved in ultraendurance exercise [4, 16, 17]; anyway, none of these examined the relationship between duration and OxS response by means of a multicomprehensive analysis. Also, differences in exercise training habits of the participants may influence the oxidation response to exercise. Therefore, the choice of a specific very homogenous experimental group of subjects, for example, elite ultraendurance athletes, is detrimental considering that, in trained individuals, antioxidant defences couple successfully with prooxidants to protect against increased oxidative stress [18, 19].

The most fundamental molecules in thiols category, for example, Cys and GSH, have been shown to limit fatigue [20] playing a critical role in circumventing exercise-induced oxidative stress and therefore one may postulate that this determines exercise performance too.

The aim of this study was to determine the redox thiols status in plasma and erythrocytes, plasmatic ROS production, total antioxidant capacity, and oxidative damage markers concentration in ultraendurance elite athletes according to the duration of a running race in order to test the presence of relationships and integration of the different components of blood redox system.

Moreover, as redox interventions that modulate ROS have the potential to improve performance, a secondary objective of this study was to investigate whether prerace antioxidant and/or thiol redox status and ROS production may be predictive of performance. This latter finding has clear implications for athletes seeking a competitive edge.

## 2. Subjects and Methods

Thirty runners were recruited and gave their consent to participate in the study. Written informed consent was signed by all participants involved after being informed of all procedures and purposes of the study. Procedures were in accordance with the Declaration of Helsinki, and institutional review board ethical approval was received. All the participants are healthy, highly trained national and international level nonprofessional endurance athletes, belonging to the Italian Ultramarathon and Trail Association (IUTA) team and consequently fully familiarized with ultramarathon running. Before basal testing, participants were asked to (a) follow three days of standardized food intake (59% carbohydrate, 29% fat, and 12% protein) avoiding any antioxidant supplementation, (b) refrain from alcohol and caffeine consumption for at least 24 h, (c) avoid every form of exercise for 48 h, and (d) visit the laboratory on the day before the ultraendurance race to have height and body weight recorded, body fat and fat-free mass determined by tetrapolar bioimpedentiometry (TBF-300A Body Composition Analyzer; Tanita Corporation, Arlington Heights, IL, USA), and one sample of blood and urine collected. A questionnaire evaluating lifestyle characteristics (i.e., smoking, alcohol drinking, and drugs habits and subjects' training experience) was also administered. The athletes, all nonsmokers, had on average 4.5 ± 2.5 years of ultraendurance experience and followed their own individual preparation programme to achieve their best results during competition. The training sessions in preparation to the race consisted of 3–5 sessions per week (about 75 ± 10.5 km/wk) and in the last week the daily running distance was 15–25 km. None of the participants had used specific antioxidant supplements during the month preceding the race, as well as any medication during the prerace week.

### 2.1. Ultramarathon Race

Participants in the study took part in a double-ring 50 km long competitive ultramarathon race, an annual event held in Seregno, Italy. The study was performed during two consecutive editions: 16 athletes were enrolled during the 2011 edition and the other 14 were enrolled in the 2012 one. The enrollment in 2012 edition was necessary in order to ensure a sample size able to support a strong statistical analysis.

Ambient temperature during the two races ranged from a minimum of 11°C to a maximum of 25°C, with relative humidity between 55 and 85%.

Participants undertook the race at their own pace and with no dietary restrictions. They consumed food (bread, fruits, and cookies) and fluids (water, beverages, and sport drinks), available at checkpoints throughout the race, ad libitum, avoiding any antioxidant supplementation. Six athletes (3 ♂ and 3 ♀) completed only one ring (50 km), eighteen (7 ♂ and 11 ♀) completed two rings (100 km), and the others retired and therefore were eliminated from this study.

### 2.2. Samples

Each athlete reported to the mobile laboratory before (pre) and immediately upon the completion (post) of the race for blood and urine samples collection. Approximately 15 mL of blood was drawn from an antecubital vein and collected in heparinized (5 mL) and EDTA (10 mL)-treated vacutainer tubes (Becton Dickinson and Company, UK). Plasma was separated by centrifuge (5702R, Eppendorf, Germany) at 3000 g for 5 min at 4°C. Samples of plasma and erythrocytes were then immediately stored in multiple aliquots at −80°C until being assayed. Also, urine samples were collected both before and after race and aliquots were stored at −80°C until analyses were performed. Samples were thawed only for the analyses, which were performed within two weeks from collection.

### 2.3. Analytical Procedure

#### 2.3.1. Thiols

Total and reduced aminothiols were measured in erythrocytes and plasma according to methods previously validated [21]. Briefly, Tris-(2-carboxyethyl)-phosphine hydrochloride (TCEP) and 4-fluoro-7-sulfamoylbenzofurazan (ABD-F) were used as reducing and derivatizing agents, respectively; reduced aminothiols were assessed by mixing erythrocytes and plasma with 10% trichloroacetic acid (1 : 1 v/v). 10 *μ*L of 0.4 M NaOH, 70 *μ*L of 1 M borate buffer, pH 11, containing 4 mM EDTA, 30 *μ*L of 1 M borate buffer, pH 9.5, containing 4 mM EDTA, and 10 *μ*L of 10 g·L^−1^ ABD-F in borate buffer (1 M, pH 9.5, containing 4 mM EDTA) were added to 100 *μ*L of each supernatant obtained. Samples were incubated at 4°C for 90 min and then 10 *μ*L was injected into the high-performance liquid chromatography (HPLC) system for analysis. Thiols separation was performed at room temperature by isocratic HPLC analysis on a Discovery C-18 column (250 × 4.6 mm ID, Supelco, Sigma-Aldrich), eluted with a solution of 0.1 mol·L^−1^ acetate buffer (pH 4.0) : methanol, 81 : 19 (v/v), at a flow rate of 1 mL·min^−1^. Fluorescence intensities were measured with excitation at 390 nm and emission at 510 nm, using a fluorescence spectrophotometer (Jasco, Japan). A standard calibration curve was used for the assayed samples. The concentration of oxidized forms was obtained by the difference between the total and reduced forms.

#### 2.3.2. EPR Measurements

An X-band EPR instrument (E-scan, Bruker BioSpin, GmbH, MA, USA) was adopted for determinations. For each recruited subject, ROS production rate was determined by means of a recently implemented EPR method [22] analyzing 50 *μ*L plasma samples treated with CMH (1-hydroxy-3-methoxycarbonyl-2,2,5,5-tetramethylpyrrolidine) probe solution (1 : 1). ROS half-life is too short compared to the EPR time scale so that they result in being EPR-invisible but become EPR detectable once “trapped” by CMH and transformed in a more stable radical species. 50 *μ*L of the obtained solution was put in a glass EPR capillary tube (Noxygen Science Transfer & Diagnostics, Germany) that was placed inside the cavity of the E-scan spectrometer for data acquisition. Acquisition parameters were microwave frequency 9.652 GHz, modulation frequency 86 kHz, modulation amplitude 2.28 G, sweep width 60 G, microwave power 21.90 mW, number of scans 10, and receiver gain 3.17·10^1^. Sample temperature was firstly stabilized and then kept at 37°C by the Temperature & Gas Controller “Bio III” unit, interfaced to the spectrometer. Spectra were recorded and analyzed by using Win EPR software (version 2.11) standardly supplied by Bruker.

EPR measurements allowed us to attain a relative quantitative determination of ROS production rate in samples. All data were, in turn, converted in absolute concentration levels (*μ*mol·min^−1^) by adopting CP^•^ (3-carboxy-2,2,5,5-tetramethyl-1-pyrrolidinyloxy) stable radical as external reference.

#### 2.3.3. Immune and Enzymatic Determinations

All the samples were assessed by immune and/or enzymatic methods using a microplate reader spectrophotometer (Infinite M200, Tecan, Austria). All the sample determinations were assessed in duplicate and the interassay coefficient of variation was in the range indicated by the kit's manufacturer.

Plasma total antioxidant capacity (TAC) was measured by an enzymatic kit (Cayman Chemical, USA). This assay is based on the ability of antioxidants present in the plasma to inhibit the oxidation of 2,2′-azinobis(3-ethylbenzothiazoline) sulfonic acid (ABTS) to the radical cation ABTS^+^ by a peroxidase. The amount of the produced ABTS^+^ has been assessed by measuring the absorbance signals at 750 nm. The antioxidants concentration is proportional to the suppression of the absorbance signal. TAC was evaluated by a trolox (6-hydroxy-2,5,7,8-tetramethylchroman-2-carboxylic acid) standard curve and was expressed as trolox-equivalent antioxidant capacity concentration (mM).

The accumulation of oxidized proteins was measured by content of reactive carbonyls. A protein carbonyl (PC) assay kit (Cayman Chemical, USA) was used to evaluate colorimetrically oxidized proteins at 370 nm. Oxidized proteins values obtained were normalized to the total protein concentration in the final pellet (absorbance reading at 280 nm), in order to consider protein loss during the washing steps, as suggested in the kit's user manual.

The measurement of thiobarbituric acid-reactive substances (TBARS) is a utilized method to detect lipid peroxidation. We used TBARS assay kit (Cayman Chemical, USA) which allows rapid photometric detection of the thiobarbituric acid malondialdehyde (TBAMDA) adduct at 532 nm. A linear calibration curve was computed from pure malondialdehyde containing reactions.

A competitive immunoassay was used for the determination of 8-isoprostane (8-iso-PGF2*α*) concentration, a marker of lipid peroxidation, in urine (Cayman Chemical, USA). Urine was purified using the solid phase extraction cartridges. The purification and the subsequent EIA assay were performed following the manufacturer's recommendations. The EIA employs 8-iso-PGF2*α* tracer and 8-iso-PGF2*α* antiserum. The sample 8-iso-PGF2*α* concentration was determined using a standard curve. Samples and standards were read at a wavelength of 412 nm.

8-OH-2-deoxyguanosine (8-OH-dG) has been established as a marker of oxidative DNA damage. This compound was quantified in excreted urine. A commercially available enzyme immunoassay EIA kit (Cayman Chemical, USA) for the measurement of 8-OH-dG was utilized. The EIA employs an anti-mouse IgG-coated plate and a tracer consisting of an 8-OH-dG-enzyme conjugate. The sample 8-OH-dG concentration was determined using an 8-OH-dG standard curve. Samples and standards were read at a wavelength of 412 nm.

Urinary concentrations of 8-iso-PGF2*α* and 8-OH-dG, as any urinary marker, vary considerably; therefore, the urinary parameters are usually standardized based on the amount of creatinine excreted in the urine when the collection of the 24 h urine is not possible. Indeed, in the absence of renal disease, the excretion rate of creatinine in an individual is relatively constant. Thus, urinary creatinine levels may be used as an index of standardization. Creatinine assay kit (Cayman Chemical, USA) was used to measure creatinine levels in urine samples. Creatinine concentration was determined using a creatinine standard curve.

### 2.4. Statistical Analysis

Statistical analysis was performed using the GraphPad Prism package (GraphPad Prism 6, GraphPad Software Inc., San Diego, CA). Data are expressed as mean ± SD. Experimental data were analyzed using repeated Shapiro-Wilk *W* test and were compared by variance analysis, ANOVA repeated measures, with Tukey's multiple comparison test to further check the among-groups significance.

A *P* value <0.05 was considered statistically significant. Pearson's product moment correlation coefficient (*R* ^2^) with 90% confidence intervals (CI) was used to examine the relationships between selected parameters: running duration and pre-post changes in erythrocytes and plasma thiols concentrations, plasma ROS production rate, TAC value, PC and TBARS concentration, and urinary 8-iso-PGF2*α* and 8-OH-dG content.

Prospective calculation of power to determine significant number was made by using the Freeware G^*∗*^Power software (http://www.psycho.uni-duesseldorf.de/abteilungen/aap/gpower3/). At a power of 80%, the calculated number of significant subjects was 15, well below the subject's population recruited for this study.

## 3. Results

No significant difference in anthropometric features, ROS production, oxidative stress biomarkers, and redox status level between 2011 and 2012 editions before race (pre) was found. Moreover, the athletes' performance (i.e., average race velocity) recorded in the two editions was not significantly different.

From the recruited runners, 6 (3 ♀ and 3 ♂) completed only one-ring race (50 km; running duration ranged from 3 h 27′ to 4 h 39′) and 18 (11 ♀ and 7 ♂) completed the two-ring race (100 km; running duration ranged from 6 h 40′ to 10 h 44′) within the time limit. The general characteristics, the anthropometric features, and the training profiles of the athletes are shown in Table 1.

Each runner's average velocity was calculated as total run distance divided by total time elapsed. The average velocity recorded was 12.96 ± 1.43 and 11.90 ± 1.60 km·h^−1^ in 50 and 100 km race, respectively.

### 3.1. Thiols

In Table 2, the values of aminothiols concentration recorded before (pre) and immediately at the end (post) of the different distance races are summarized. No significant difference in aminothiols concentration at prerace between the two groups of athletes was observed.

In plasma, significant changes were detected only after 100 km race in total (*P* < 0.0001), reduced (*P* < 0.0001), and oxidized (*P* < 0.0001) Cys; total (*P* < 0.0001) Hcy; total (*P* < 0.0001), reduced (*P* < 0.05), and oxidized (*P* < 0.0001) CysGly; and total (*P* < 0.0001) and oxidized (*P* < 0.0001) GSH concentrations.

After the ultraendurance race, a significant (*P* < 0.01) change between pre- and postrace in erythrocytes was observed only in oxidized GSH concentration after 100 km race.

**Figure 1:**
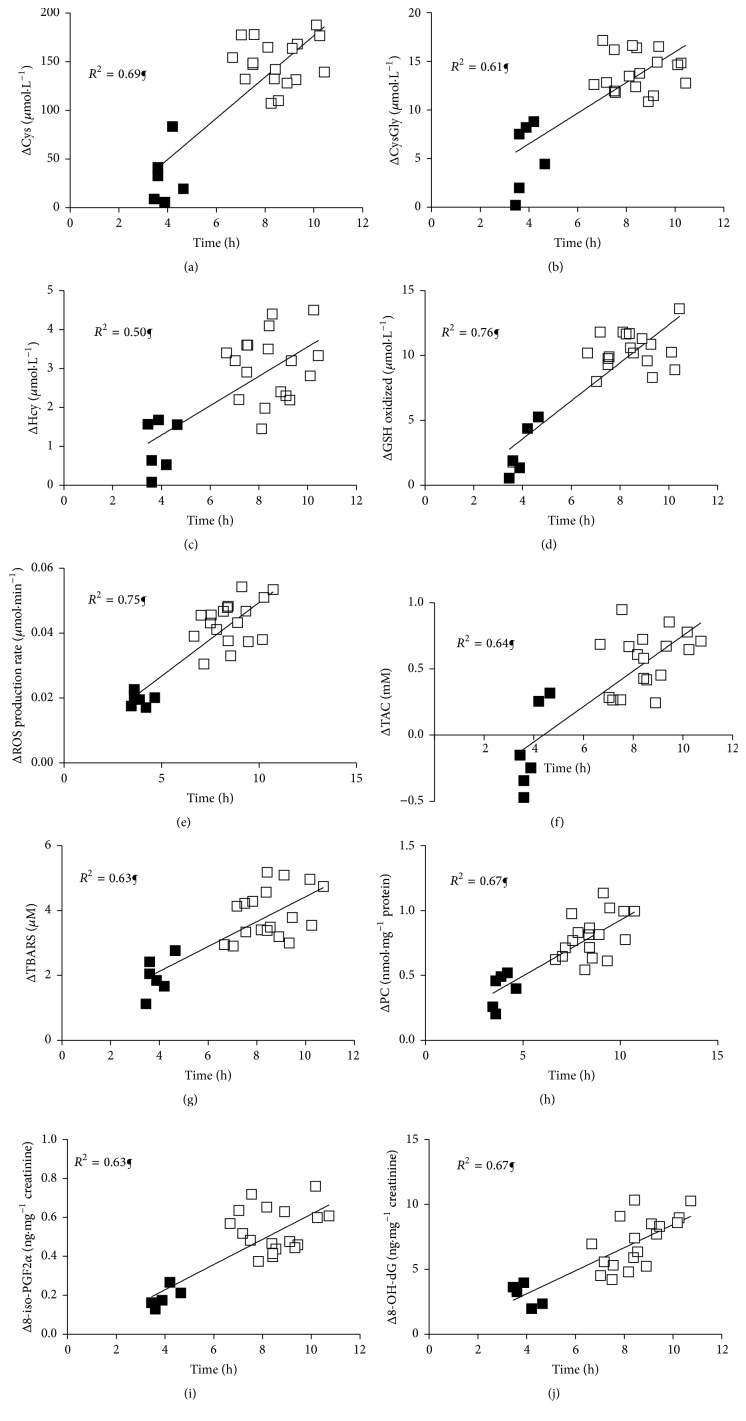
Panel plots of the relationship between individual duration of race (h) and relative Δ of total (a) Cys (*μ*mol·L^−1^), (b) CysGly (*μ*mol·L^−1^), (c) Hcy (*μ*mol·L^−1^), (d) oxidized GSH (*μ*mol·L^−1^), (e) ROS production rate (*μ*mol·min^−1^), (f) TAC (mM), (g) TBARS (*μ*M), (h) PC (nmol·mg^−1^ protein), (i) 8-iso-PGF2*α* (ng·mg^−1^ creatinine), and (j) 8-OH-dG (ng·mg^−1^ creatinine) recorded in 50 km (full symbols) and in 100 km (empty symbols) race are shown. The linear regression fit (solid line) and the correlation coefficient (*R* ^2^) reported for each relationship are shown too. Significant linear relationships, *P* < 0.0001 (¶ symbol), were estimated.

The difference (Δ) between post- and prerace concentrations was plotted for total Cys, CysGly, Hcy, and oxidized GSH values observed in plasma of each examined participant as a function of individual race time (Figures 1(a), 1(b), 1(c), and 1(d) resp.); a significant (*P* < 0.0001) positive correlation (*R* ^2^ = 0.69; *R* ^2^ = 0.61; *R* ^2^ = 0.50; and *R* ^2^ = 0.76, resp.) was observed.

### 3.2. Oxidative Stress Biomarkers

Concentrations of the oxidative damaged biomarkers recorded before (pre) and immediately at the end (post) of the race in all athletes running the different distances examined are shown in Table 3.

No significant differences were observed at prerace in the athletes of both groups for all evaluated parameters.

A significant increase of plasmatic ROS production rate (+12%: *P* < 0.001 versus +29%: *P* < 0.0001 in 50 and 100 km, resp.) was observed at postrace. After competition, TAC concentration demonstrated a different pattern in the different groups of athletes. Indeed, in 50 km group, a nonsignificant decline from pre- to postrace values was observed. On the contrary, at postrace in the 100 km group a significant (*P* < 0.0001) increase in TAC value was observed.

PC values significantly increased after race and the amount was different in the two groups with higher values after the longer competition (+54%: *P* < 0.05 versus +115%: *P* < 0.0001 for 50 and 100 km, resp.). The same trend was observed for TBARS concentration. Nevertheless, in this case, the significant increase at postrace according to the running distance was less evident (+28%: *P* < 0.05 versus +55%: *P* < 0.001 for 50 and 100 km, resp.).

A significant increase at postrace, in all groups according to the different competition distance, in the mean urinary excretion levels of both 8-iso-PGF2*α* (+43%: *P* < 0.05 versus +135%: *P* < 0.001) and 8-OH-dG (+71%: *P* < 0.05 versus +158%: *P* < 0.0001) for 50 and 100 km, respectively, was observed too.

The difference (Δ) between post- and prerace concentrations for ROS production rate, TAC, TBARS, PC, 8-iso-PGF2*α*, and 8-OH-dG values, for each examined participant, as a function of individual running time was plotted in Figure 1. A significant (*P* < 0.0001) positive correlation was observed between ΔROS production (*R* ^2^ = 0.75, Figure 1(e)), ΔTBARS (*R* ^2^ = 0.63, Figure 1(g)), ΔPC (*R* ^2^ = 0.67, Figure 1(h)), and the race time. The difference (Δ) in TAC values recorded before and after the race resulted to be in some athletes negative after the 50 km competition but always positive after 100 km race. A significant positive correlation (*P* < 0.0001, *R* ^2^ = 0.64, Figure 1(f)) between ΔTAC individual values and the correspondent race time was observed. Also, a significant (*P* < 0.0001) direct linear relationship was observed between both urinary Δ8-iso-PGF2*α* (*R* ^2^ = 0.63, Figure 1(i)) and Δ8-OH-dG (*R* ^2^ = 0.67, Figure 1(j)) individual values and the race time.

### 3.3. ROS Production Rate Relationships

**Figure 2:**
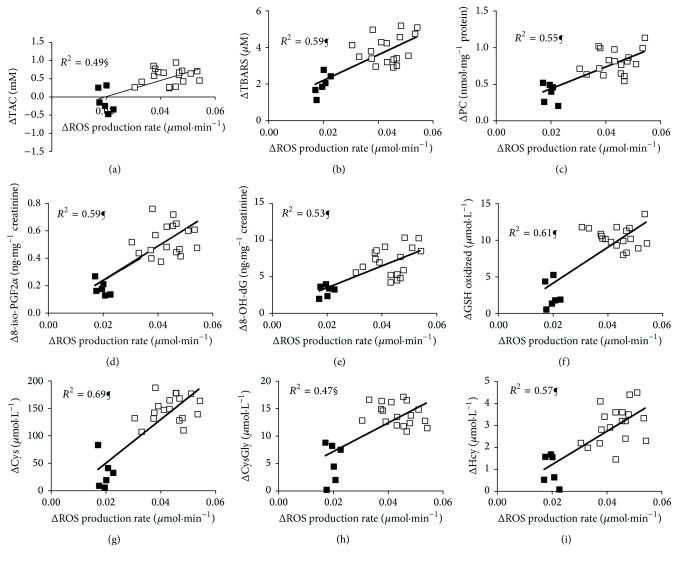
The relationships between individual plasmatic ΔROS production rate (*μ*mol·min^−1^) and Δ of (a) TAC (mM), (b) TBARS (*μ*M), (c) PC (nmol·mg^−1^ protein), (d) 8-iso-PGF2*α* (ng·mg^−1^ creatinine), (e) 8-OH-dG (ng·mg^−1^ creatinine), (f) oxidized glutathione (*μ*mol·L^−1^), and total (g) Cys (*μ*mol·L^−1^), (h) CysGly (*μ*mol·L^−1^), and (i) Hcy (*μ*mol·L^−1^) recorded in 50 km (full symbols) and in 100 km (empty symbols) race are shown. The linear regression fit (solid line) and the correlation coefficient (*R* ^2^) reported for each relationship are shown too. Significant linear relationships, *P* < 0.001 (§ symbol) and *P* < 0.0001 (¶ symbol), were estimated.

The individual ΔTAC levels significantly increased directly related (*P* < 0.001, *R* ^2^ = 0.49, Figure 2(a)) to the increase of correspondent ΔROS production rates.

Analogously, in all athletes examined, it was apparent that all changes indicating increased ROS production were directly related to an increase in oxidative damage biomarkers concentration (Figure 2). In detail, the individual Δ of TBARS (*R* ^2^ = 0.59, Figure 2(b)), PC (*R* ^2^ = 0.55, Figure 2(c)), 8-iso-PGF2*α* (*R* ^2^ = 0.59, Figure 2(d)), and 8-OH-dG (*R* ^2^ = 0.53, Figure 2(e)) levels significantly (*P* < 0.0001) increased according to the increase of correspondent ΔROS production rates. The individual Δ of oxidized GSH (*P* < 0.0001, *R* ^2^ = 0.61, Figure 2(f)) and total Cys (*P* < 0.0001, *R* ^2^ = 0.69, Figure 2(g)), CysGly (*P* < 0.001, *R* ^2^ = 0.47, Figure 2(h)), and Hcy (*P* < 0.0001, *R* ^2^ = 0.57, Figure 2(i)) levels significantly increased directly related to the increase of correspondent ΔROS production rates.

**Figure 3:**
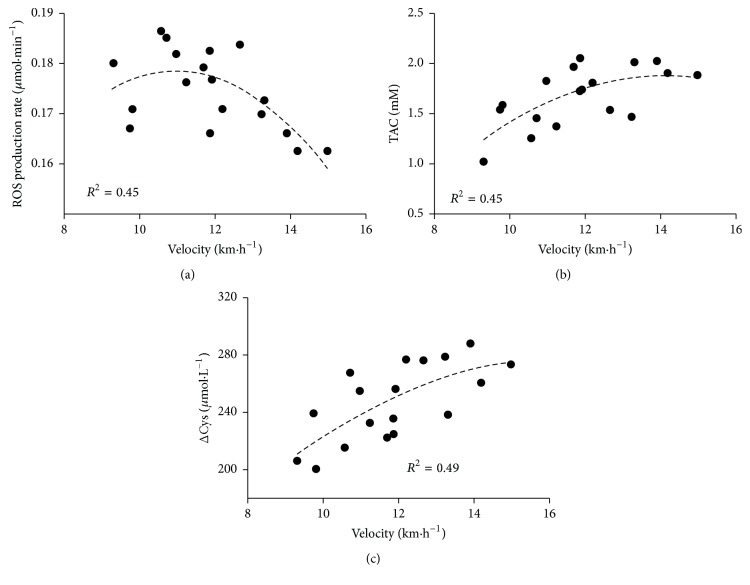
The relationships between individual mean velocity (km·h^−1^) and relative prerace (a) ROS production rate (*μ*mol·min^−1^), (b) TAC (mM), and (c) total Cys (*μ*mol·L^−1^) concentration recorded in 100 km race are shown. The polynomial regression fit (dashed line) and the correlation coefficient (*R* ^2^) reported for each relationship are shown too.

The relationship between prerace ROS production rates (Figure 3(a)), TAC levels (Figure 3(b)), total Cys concentrations (Figure 3(c)), and mean velocity sustained by every single athlete during the 100 km race was shown too.

## 4. Discussion

Exercise is able to induce alterations in the redox homeostasis which increase when the intensity and the duration of exercise increase [23, 24]. The findings of this study present the relationship between OxS response and duration of a competitive race. As far as we know, this is the first study investigating this theme in prolonged strenuous exercise (>4 h), using only elite ultraendurance athletes (see Table 1 for athlete's profile), in order to increase the sensitivity of the study reducing the sample's heterogeneity (introduced by different individual training habits of participants), and examining a multicomprehensive setting of parameters particularly ROS production in plasma, determined by Electron Paramagnetic Resonance and redox thiols status in erythrocytes and plasma. Generally, ultraendurance exercise causes an increase of OxS, which, despite being alleviated in a matter of hours or days depending on the biomarker assessed [25, 26], anyway induces the generation of oxidative damage that may be potentially relevant in giving rise of clinical alterations to redox homoeostasis [4, 15, 27].

Thereafter, endurance exercise may be considered as a model of physiological situation able to promote the deleterious actions of ROS in plasma and into blood cells.

Moreover, the present study examined the dynamic relation existing between the reduced-oxidized forms of various aminothiols, the major nonenzymatic antioxidants in human plasma, and erythrocytes. Thiol groups located on various molecules are central in redox-sensitive cell signaling mechanisms [28] by means of a reversible process representing a regulated functional switch. Specifically, changes in redox state of critical thiols in receptors, enzymes, transcription factors, or transport systems are thought to function in control of fundamental processes such as gene expression, cell proliferation, or apoptosis [6, 28]. For all these reasons, thiols play a fundamental role in cell biology, biochemistry, and pharmacology. Therefore, simultaneous determination of aminothiols may be a useful tool in studying oxidative stress and metabolic and redox regulation. After the 100 km, plasma GSH concentration was unchanged, as reported previously [11]. Anyway, plasma-oxidized glutathione concentration exhibited a large increase related to the other examined thiols' (i.e., Cys, CysGly, and Hcy) perturbation (Table 2 and Figure 1).

Previously [29], it was postulated that duration and intensity of exercise are relevant factors for the modulation of Hcy. The present data confirmed (Figure 1(c)) and amplified this observation showing a significant increase also in plasma total Cys (Figure 1(a)) and CysGly (Figure 1(b)) concentrations according to race duration. This is important as clinical and epidemiological evidence suggested that the plasmatic level of Hcy, though only moderately elevated, is associated with cardiovascular disease [7].

Erythrocytes are much more vulnerable to oxidative damage during intense exercise because of their continuous exposure to high oxygen fluxes and their high concentrations of polyunsaturated fatty acids and heme iron [30]. The erythrocytes' chronic alteration, derived by oxidative damage, is currently viewed as potent inductor of disease or morbidity [8]. However, our data showed that even if significant perturbation of thiols redox status in plasma was demonstrated, a significant increase in oxidized GSH was observed in erythrocytes only in the heaviest exercise conditions (i.e., 100 km) suggesting that duration and intensity of exercise are determinant in the imbalance of their redox status. Anyway, we must underline the notion that aminothiols balance was preserved probably because of the enhancement of defense system induced by the endurance-trained status of the examined athletes [31]. The insufficient antioxidant response in the plasmatic compartment where oxidized GSH levels increase directly related to the increase of ROS production (Figure 2(f)) is perhaps due to the appreciably lower thiols concentration/capacity in plasma than in erythrocytes and/or mechanisms apt to prefer the preservation of redox status in cellular compartments [31].

Oxidative damage after physical exercise is directly related to the type, intensity, and duration of exercise [23, 24, 32]. Thus, it seems that different forms of exercise resulted in different levels of oxidative damage, although data reporting the exact influence of the duration of the specific exercise examined (i.e., long distance running) on levels of OxS was never reported before.

**Figure 4:**
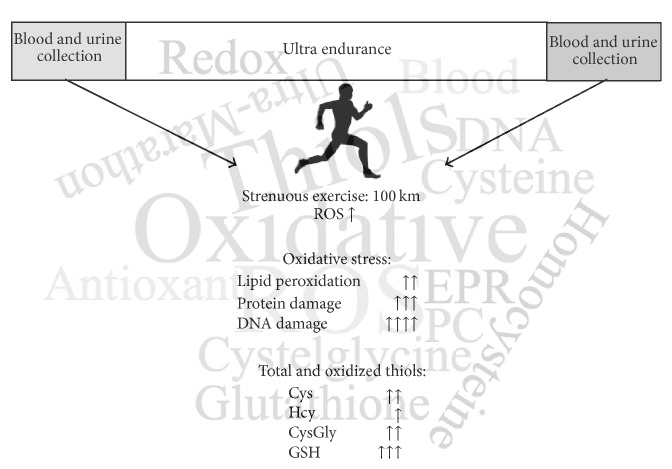
Scheme of the influence of strenuous exercise (100 km) on ROS production, oxidative stress, and thiol oxidation. Evaluation by plasma and urine collection.

Participants in this study that completed an ultramarathon, with average duration longer than 4 hours, stimulated a significantly higher proportion of various markers of oxidative damage compared to prerace (Figure 4). Indeed, a substantial amount of ROS was generated and, consequently, the potential damage to lipids, proteins, and DNA may be greater [33]. The results of works reported in the literature, which examined the effects of competitive ultraendurance exercise on various markers of oxidative damage, showed no permanent damage [11, 25, 26, 34]. Therefore, in the present study, blood sampling was limited to the time immediately after the race.

The 50 km race increased ROS production and consumed antioxidants as reflected in the nonsignificant decrease of TAC (see Table 3). Otherwise, the significant TAC increase observed after 100 km race means that exercise activated some of the body's antioxidant mechanisms in plasma [35]. Mobilization of tissue antioxidant stores into plasma is probably one of the mechanisms responsible for the marked increase observed. This is a widely accepted phenomenon that would help to maintain or even increase plasma antioxidant status when needed [36]. The significant increase in plasmatic total thiols content (Table 2) may suggest a confirmation of this concept.

The PC content is the most general and widely used marker of severe oxidative protein damage. An exercise bout of moderate intensity, but long duration (ultraendurance running), has been reported to elicit immediate and prolonged postexercise increases in plasma PC concentration [11, 37]. The significant increase in carbonyl content after race is directly related to the individual running duration (Figure 1(h)).

The observed overall increase in PC level in response to prolonged duration exercise may result from the formation of new PC groups outnumbering proteolytic clearance, by means of proteasome system, of those present at baseline. The proteasome system is an organized assembly of proteins present in all cell types and in plasma that functions to degrade irreversibly modified proteins, such as those containing carbonyl groups. However, Wadley et al. [38] proposed that proteasome activity may be reduced after prolonged duration exercise, due to ROS-induced inactivation of the functional proteasome which cleaves PC groups in plasma directly. Data reported in Figure 2(c) are in accordance with this hypothesis.

The observed increase in TBARS levels supports the hypothesis that the ultramarathon runners have increased lipid peroxidation and this is in accordance with other similar studies [11] in which OxS was evaluated by using the same biomarker. The use of TBARS to detect lipid peroxidation in humans has been criticized due to a lack of accuracy and validity of this method; anyway, the recorded increased levels of urinary 8-iso-PGF2*α*, a more reliable marker to assess the lipid peroxidation [39], are in agreement with TBARS production trend and with measurements of Knez et al. [40], Mastaloudis et al. [16], and Neubauer et al. [25]. Also, for the increase of the two lipid peroxidation biomarkers examined, a direct relationship with the individual running duration was observed.

In the present study, as reports elsewhere [11], ultraendurance exercise caused an increase in damage to DNA too. This latter is reported to be linked to increased cancer and cardiovascular disease risk [14] and to atherosclerotic plaques [13]. Moreover, elevated levels of DNA damage might increase infection risk after endurance exercise events [41] because of damage to lymphocytes that might inhibit cell-mediated immunity. The significant increase in DNA damage biomarker concentration after race was directly related to the individual running duration.

It is apparent that all changes indicating increased ROS production are directly related to an increase in oxidative stress biomarkers concentration (Figure 2) and higher ROS production levels correspond to higher oxidative damage biomarker production. These results confirm previous observations [22, 42] reporting analogous relationships determined at rest condition. Accumulating evidence suggests that ROS generated during exercise modulate generation pathways of oxidative damage and antioxidant-redox compounds. The strength of the correlations among the measured variables reflects their degree of interdependency.

A secondary objective of this study was to investigate whether antioxidant status might be predictive of physical performance. The observed relationship between prerace ROS production rate (Figure 3(a)), TAC level (Figure 3(b)), total Cys concentration (Figure 3(c)), and individual mean velocity sustained during 100 km race indicates that the former parameters registered at baseline may perhaps predict running performance.

Observing in more detail the distribution of the experimental data, the relationships between performance and redox status lead to the hypothesis that there is hormetic relationship between prerace ROS production rate and mean velocity. A similar specular trend was observed for total antioxidant capacity and total Cys concentration and mean velocity. Elevated endogenous antioxidant capacity may enable buffering of ROS production, subsequently reducing the magnitude of oxidative stress biomarker formation during exercise according to the individual training degree. In this proposed model, it is assumed that the muscle redox state is a physiologically regulated variable balanced by matching the rates of ROS production with cellular antioxidant buffering capacity. The paradigm predicts that an optimal cellular redox state exists whereby conditions are ideal for exercise performance. Generally, the dichotomy concept between good and evil is believed: “free radicals are evil and antioxidants are good.” This concept has fueled widespread appetite for antioxidant-rich foods and antioxidant supplements and athletes are loaded up with the belief that antioxidants improve performance even if this has never been experimentally confirmed. The hormetic relationship hypothesis according to a growing body of literature suggests otherwise.

Moreover, it is reported that the body, by means of training procedures, has the ability to increase its antioxidant capacity in order to control OxS during exercise [18, 43]. A previous study [19], comparing the participants' individual data before, during, and after training, indicated convergence of TAC values towards an optimal level induced by training procedure. Therefore, we might postulate that runners that had reached this optimal level, by means of their own training protocol, were the subjects predisposed to reach the highest mean velocity during the race. However, we want to emphasize that this concept is highly speculative and has not been proven yet. Indeed, unfortunately, the number of the examined subjects in this study was relatively low reducing the statistical power to establish such hypothesis.

## 5. Limitations

The authors are aware that this “field” setting suffers from certain limitations. There was no chance of calling back the participants, coming from all over Italy, in order to extend the study to the postrace recovery. Therefore, mechanisms finding how oxidative stress is implicated in the recovery phase and chronic clinical implications were not included in this study limited to the acute observation.

The food intake of the athletes during the race was not standardized; therefore, its contribution to the antioxidant capacity cannot be assessed. However, the observed increase of TAC was very important and cannot be attributed only to the antioxidants included in food and drinks.

As positive remark, we would underline the notion that the choice of assessing a competitive race may ensure a very truthful experimental condition offering extremely motivated subjects too.

## 6. Conclusions

This study confirms that ultraendurance very prolonged (>4 hours) exercise caused an increase of plasmatic ROS production and oxidative stress and perturbation of aminothiols redox status. We reported that, independently of the specific biomarker examined, the increase is always linearly and directly related to the duration of exercise and possibly to the individual fitness level. The strength of the correlations among measured variables reflects their degree of interdependency. Nevertheless, the redox status of erythrocytes was substantially unchanged suggesting preservation of cellular and tissue equilibrium. This would imply that mechanisms regulating blood oxidative status are conserved across those different stages that demand strong increases in physical activity. Thiol antioxidants appear to improve performance during endurance exercise. A predictive role of the individual running performance might be probably attributable to resting prerace level of both ROS production and TAC and Cys levels.

## Figures and Tables

**Table 1:** Mean (±SD) of the general characteristics, the anthropometric features, and the training profiles collected from the athletes taken altogether at prerace.

Parameter	50 km (*n* = 6)	100 km (*n* = 18)
Age (years)	41.83 ± 5.98	41.44 ± 3.61
Weight (kg)	59.86 ± 10.35	58.55 ± 10.44
Height (m)	1.67 ± 0.08	1.68 ± 0.10
Body mass index (kg·m^−2^)	21.08 ± 2.11	20.44 ± 1.79
Fat mass (%)	7.92 ± 1.51	8.54 ± 4.01
Fat-free mass (kg)	55.02 ± 8.93	53.72 ± 10.91
km run per wk	75 ± 10.5	
Time per wk	5 h 25′ ± 25′	

**Table 2:** Mean (±SD) of the redox status in plasma and erythrocytes of aminothiols in ultramarathon athletes before (pre) and immediately after (post) 50 and 100 km race. Concentrations of various forms are expressed in *μ*mol·L^−1^. Significant differences compared to prerace: *P* < 0.05 (*∗* symbol), *P* < 0.01 (# symbol), and *P* < 0.0001 (¶ symbol).

	50 km				100 km		
	Pre	Post	*P*		Pre	Post	*P*
Plasma							
*Cysteine*							
Total	266.95 ± 25.06	298.85 ± 27.00	ns		247.15 ± 26.92	396.58 ± 40.02	¶
Reduced	10.05 ± 2.08	12.93 ± 0.79	ns		10.76 ± 2.89	15.33 ± 2.66	¶
Oxidized	256.90 ± 24.58	285.93 ± 26.62	ns		234.52 ± 25.94	381.25 ± 38.97	¶
*Homocysteine *							
Total	6.03 ± 1.34	7.04 ± 1.85	ns		6.41 ± 1.58	9.47 ± 1.81	¶
Reduced	0.05 ± 0.03	0.05 ± 0.02	ns		0.08 ± 0.03	0.06 ± 0.03	ns
*Cysteinylglycine*							
Total	24.75 ± 2.61	29.96 ± 3.67	ns		24.25 ± 3.49	38.21 ± 3.54	¶
Reduced	2.00 ± 0.65	2.11 ± 0.52	ns		2.35 ± 0.40	1.92 ± 0.45	*∗*
Oxidized	22.76 ± 2.52	27.85 ± 3.25	ns		22.03 ± 3.57	36.29 ± 3.65	¶
*Glutathione*							
Total	7.12 ± 1.47	9.66 ± 1.45	ns		7.20 ± 1.12	17.63 ± 2.23	¶
Reduced	0.41 ± 0.08	0.41 ± 0.08	ns		0.44 ± 0.08	0.44 ± 0.10	ns
Oxidized	6.71 ± 1.44	9.25 ± 1.50	ns		6.76 ± 1.24	17.19 ± 1.84	¶

Erythrocyte							
*Cysteine*							
Total	78.46 ± 7.99	87.01 ± 7.62	ns		78.84 ± 10.43	84.53 ± 11.69	ns
Reduced	12.42 ± 1.69	12.44 ± 1.69	ns		12.02 ± 2.37	11.80 ± 2.71	ns
Oxidized	66.04 ± 7.77	74.57 ± 7.25	ns		67.49 ± 8.59	72.73 ± 11.69	ns
*Homocysteine*							
Total	2.35 ± 0.51	2.39 ± 0.33	ns		2.36 ± 0.47	2.44 ± 0.32	ns
Reduced	0.90 ± 0.13	0.84 ± 0.22	ns		0.89 ± 0.18	0.86 ± 0.26	ns
Oxidized	1.45 ± 0.43	1.55 ± 0.36	ns		1.47 ± 0.42	1.58 ± 0.23	ns
*Cysteinylglycine*							
Total	2.15 ± 0.35	2.23 ± 0.31	ns		2.26 ± 0.34	2.21 ± 0.29	ns
Reduced	0.35 ± 0.07	0.44 ± 0.23	ns		0.33 ± 0.12	0.32 ± 0.10	ns
Oxidized	1.80 ± 0.30	1.78 ± 0.34	ns		1.94 ± 0.28	1.90 ± 0.29	ns
*Glutathione*							
Total	1666.88 ± 90.44	1704.13 ± 113.41	ns		1667.08 ± 132.21	1692.80 ± 131.13	ns
Reduced	1482.12 ± 82.21	1450.11 ± 62.13	ns		1489.33 ± 132.16	1401.32 ± 122.26	ns
Oxidized	184.77 ± 91.10	254.01 ± 95.65	ns		178.70 ± 54.57	291.48 ± 76.41	#

**Table 3:** Mean (±SD) of the oxidative stress biomarkers concentrations in plasma and urine of ultramarathon athletes before (pre) and immediately after (post) 50 and 100 km race. Significant differences compared to prerace: *P* < 0.05 (*∗* symbol), *P* < 0.001 (§ symbol), and *P* < 0.0001 (¶ symbol).

	50 km				100 km		
	Pre	Post	*P*		Pre	Post	*P*
Plasma							
ROS (*μ*mol·min^−1^)	0.16 ± 0.01	0.18 ± 0.01	§		0.17 ± 0.01	0.22 ± 0.01	¶
TAC (mM)	1.80 ± 0.21	1.70 ± 0.15	ns		1.68 ± 0.29	2.25 ± 0.20	¶
PC (nmol·mg^−1^ protein)	0.71 ± 0.19	1.10 ± 0.21	*∗*		0.69 ± 0.16	1.49 ± 0.26	¶
TBARS (*μ*M)	6.93 ± 1.61	8.91 ± 1.70	*∗*		7.05 ± 1.28	10.95 ± 1.53	¶

Urine							
8-Iso-PGF2*α* (ng·mg^−1^ creatinine)	0.42 ± 0.13	0.60 ± 0.14	*∗*		0.40 ± 0.13	0.94 ± 0.13	§
8-OH-dG (ng·mg^−1^ creatinine)	4.38 ± 1.16	7.48 ± 1.16	*∗*		4.50 ± 0.94	11.61 ± 1.80	¶
